# Correlation analysis between physicians' evaluations of doctor–patient relationship and their preferences for shared decision-making in China

**DOI:** 10.3389/fpsyt.2022.946383

**Published:** 2022-10-05

**Authors:** Zhuo-Ran Chen, Li Zhang, Ya-Wei Chen, Meng-Yang Xu, Hang Jia, Meng-Ying Li, Yu-Han Lou, Ling Lan

**Affiliations:** ^1^Henan No.3 Provincial People's Hospital, Zhengzhou, China; ^2^The Third People's Hospital of Zhengzhou, Zhengzhou, China; ^3^GeneCast Biotechnology Co., Ltd., Beijing, China; ^4^Hunan Aerospace Hospital, Changsha, China; ^5^Nanyang City Center Hospital, Nanyang, China; ^6^Kaifeng Central Hospital, Kaifeng, China; ^7^Henan Provincial People's Hospital, Zhengzhou, China; ^8^People's Hospital of Zhengzhou University, Zhengzhou, China; ^9^People's Hospital of Henan University, Zhengzhou, China

**Keywords:** shared decision-making, doctor–patient relationship, physician, China, patient decision aid

## Abstract

Shared decision-making (SDM) is a scientific and reasonable decision-making model. However, whether physicians choose SDM is usually influenced by many factors. It is not clear whether the strained doctor–patient relationship will affect physicians' willingness to choose SDM. Through a survey by questionnaire, 304 physicians' evaluations of doctor–patient relationship (DPR) were quantified by the difficult DPR questionnaire-8. Their preferences for SDM and the reasons were also evaluated. The correlation between physicians' evaluations of DPR and their preferences for SDM were analyzed. 84.5% physicians perceived DPR as poor or strained, 53.3% physicians preferred SDM, mainly because of the influences of medical ethics and social desirability bias. Their preferences for SDM were not significantly correlated with their evaluations of DPR (*P* > 0.05). Physicians with different evaluations of DPR (good, poor, and strained) all had similar preferences for SDM (42.6, 56.4, and 42.9%), with no significant difference (*P* > 0.05). There was no correlation between physicians' evaluations of DPR and their preferences for SDM. Physicians' evaluations of poor DPR did not affect their preferences for SDM. This may be influenced by the medical ethics and social desirability bias.

## Introduction

Shared decision-making (SDM) is an advocated clinical decision-making model in which physicians and patients work together to decide on clinical diagnoses and treatment plans. In SDM, a physician informs a patient about the efficacy, benefits and risks of a treatment plan; the patient informs the physician about his/her views and concerns about the disease and related risks; and finally, the physician and patient make right and reasonable choice together regarding issues related to diagnosis and treatment during the medical process (1–5). SDM requires physicians to consider the best scientific evidence and the values, goals and propensities of patients (6). As a good clinical decision-making model, SDM reflects the patient-centered approach, which is more conducive to ensuring individualized diagnosis and treatment, reducing medical risks and improving patient compliance and therapeutic effects (2, 3, 7–15) than the traditional informational model (in which physicians inform patients of the pros and cons of the available treatment options and let the patients make the final decision alone) and the paternalistic model (in which physicians make all decisions on behalf of the patients). Therefore, SDM has received increasing attention from both physicians and patients in various specialties in many countries including psychiatrists (16, 17). Salyers and Zisman-Ilani (17) pointed out that SDM required patient involvement in mental health service delivery and in promoting mental well-being. In recent years, patient decision aid (PDA) has become one of the effective ways of SDM. It can take many forms, such as tables, movies, or applications. For example, Ottawa Personal Decision Guide, Annalisar software, etc. (18).

On the other hand, good DPR is the necessary foundation for the smooth development of medical activities, which can not only promote patients' recovery, but also be very important for physicians' mental health. However, in China, the tense doctor–patient relationship (DPR) has become a general problem. A data published in 2019 by China Medical Association showed that there were 90,000 medical disputes in China in 2016, in which there were above 50% of medical staff subjected to verbal violence and 15% of the medical staff suffered physically hurt (19). Under the influence of tense DPR, physicians are prone to develop a sense of identity and alertness, increase psychological pressure and subjectively cause a poor evaluation of DPR. The constant deterioration of DPR in recent years has manifested as a lack of understanding and trust between physicians and patients (20–26).

As one of the key subjects who choice SDM, physicians' preference for SDM is crucial to promoting its development. However, under the current tense situation of DPR in China, physicians' evaluations of DPR might affect their diagnosis and treatment behaviors, such as fear of responsibility, conservative treatment and unwillingness to communicate, which should also include the impact on whether they are willing to choose SDM. In the context of increasingly strained DPR, what are physicians' preferences for SDM? Whether is there correlation between physicians' evaluations of DPR and their preferences for SDM? No relevant research has been reported. Therefore, this study analyzed physicians' preferences for SDM and reasons, and the correlation between physicians' evaluations of DPR and their preferences for SDM.

## Materials and methods

### Participants

329 clinical physicians were selected randomly as study participants from 22 clinical departments in a general hospital in China. A total of 329 questionnaires were distributed, and 312 questionnaires were recovered on site. A total of 304 (92.4%) valid questionnaires were included in this study. All participants gave their informed consent for inclusion before they participated in the study. The study was conducted in accordance with the Declaration of Helsinki, and the protocol was approved by the Ethics Committee of Zhengzhou University in China.

### Physicians' evaluations of DPR

The Difficult DPR Questionnaire-10 (DDPRQ-10) (27, 28) was widely used to quantitatively evaluate physicians' evaluations of DPR. Yang H et al. revised it to form a Chinese-specific DDPRQ-8, which had good reliability and validity (29). The contents and scoring methods of DDPRQ-8 was respectively shown as Table 1. The evaluations of DPR were classified into three levels based on the DDPRQ-8 score: good (0–16 points), poor (17–32 points), and strained (33–48 points).

**Table 1:** The difficult doctor–patient relationship questionnaire-8 (DDPRQ-8) for physicians.

**Contents**	**Scores**					
Do you want to visit your patients next time after you see them today?	1	2	3	4	5	6
Do you find that some of your patients are frustrated?	1	2	3	4	5	6
Do your patients want to control your decision about disease diagnosis and treatment?	1	2	3	4	5	6
Are you annoyed or frustrated by your patients' vague complaints about their diseases?	1	2	3	4	5	6
Do your patients have a tendency to abandon themselves?	1	2	3	4	5	6
Do you find yourself secretly (privately) wishing your patients would not come back?	1	2	3	4	5	6
Do you think it's time consuming to take care of your patients?	1	2	3	4	5	6
Are you having trouble communicating with your patients?	1	2	3	4	5	6

1, never; 2, no; 3, a little; 4, yes nearly; 5, yes; 6, yes strongly.

### Physicians' preferences for SDM and the reasons

The open ended questions in questionnaires were used. Physicians' preferences for SDM were classified into three levels and five categories as followed: high propensity (including considerably high propensity and relatively high propensity), neutral propensity, and low propensity (including relatively low propensity and extremely low propensity). Additionally, the reasons of physicians' preferences for SDM were investigated, including the influence of medical ethics (presence or absence), social desirability bias (presence or absence), DPR (good or poor), patients' willingness to participate in decision-making (strong or weak), patients' level of medical knowledge (high or low), and whether SDM is necessary for patient's conditions (yes or no). Three levels, five categories and six reasons of SDM preference were listed on the questionnaire. Data was collected by allowing physicians to self-evaluate and choose an appropriate level, category and reason respectively.

### Statistical analysis

The sample size was calculated by a formal power analysis. The significance level of α was set to 0.05. Fisher's exact test showed that the sample size was at least 200, which can provide >80% potency to detect the differences. SPSS 24.0 was used for data analysis. Chi-square test was used to compare the physicians' evaluations of DPR under the demographic information, and analyze the difference of the reasons of physicians' preferences for SDM. Spearman's rank correlation coefficient and Fisher's exact test were used to analyze the correlation between physicians' evaluations of DPR and their preferences for SDM. *P* < 0.05 was considered statistically significant.

## Results

### Demographic information

The demographic information of physicians included department, age, gender, educational level, and professional title. 304 physicians came from 22 clinical departments including neurology, psychiatry, gastroenterology, hepatology, respiratory and pediatrics, etc. (Table 2). There were 64.5% male and 35.5% female participants, with an average age of 38.8 ± 8.9 years (range: 24–60 years). They included 36 (11.8%) resident physicians, 126 (41.4%) attending physicians, 68 (22.4%) associate chief physicians, and 74 (24.3%) chief physicians. The educational backgrounds of participants were as followed: 55 Bachelors (18.1%), 166 Masters (54.6%) and 83 Doctors (27.3%) (Table 3).

**Table 2:** The department distribution of physicians.

**Department**	**Physician**	
	***n***	***n(%)***
Neurology	9	2.96
Psychiatry	7	2.30
Gastroenterology and hepatology	16	5.26
Respiratory	13	4.28
Cardiovascular	13	4.28
Nephropathy and rheumatism	12	3.95
Hematology	15	4.93
Endocrine	15	4.93
Oncology	15	4.93
Gastrointestinal surgery	9	2.96
Thyroid and mammary surgery	18	5.92
Urology surgery	15	4.93
Hepatobiliary surgery	14	4.60
Orthopeadic surgery	10	3.29
Thoracic surgery	11	3.62
Neurosurgery	12	3.95
Cardiac surgery	14	4.60
Obstetrics and gynecology	19	6.25
Pediatrics	14	4.60
Otorhinolaryngology	13	4.28
Ophthalmology	13	4.28
Emergency	16	5.26
Intensive care unit	11	3.62

**Table 3:** Physicians' evaluations of doctor–patient relationship (DPR) under the demographic information.

**Demographic information**		***n*** **(%)**	**Evaluation of DPR [*****n*** **(%)]**		
			**Good**	**Poor**	**Strained**
**Age (years)**	≤ 30	67 (22.0%)	9 (13.4%)	54 (80.6%)	4 (6.0%)
	31–40	117 (38.5%)	12 (10.3%)	95 (81.2%)	10 (8.5%)
	41–50	78 (25.7%)	15 (19.2%)	61 (78.2%)	2 (2.6%)
	≥51	42 (13.8%)	11 (26.2%)	26 (61.9%)	5 (11.9%)
		*X* ^2^	7.183	7.193	3.760
		*P*	0.066	0.066	0.276
**Gender**	Male	196 (64.5%)	31 (15.8%)	153 (78.1%)	12 (6.1%)
	Female	108 (35.5%)	16 (14.8%)	83 (76.9%)	9 (8.3%)
		*X* ^2^	0.053	0.059	0.529
		*P*	0.817	0.809	0.467
**Educational background**	Bachelor	55 (18.1%)	8 (14.5%)	39 (70.9%)	8 (14.5%)
	Master	166 (54.6%)	22 (13.3%)	134 (80.7%)	10 (6.0%)
	PhD/MD	83 (27.3%)	17 (20.5%)	63 (75.9%)	3 (3.6%)
		*X* ^2^	2.255	2.488	6.591
		*P*	0.324	0.288	0.037
**Professional title**	Resident physician	36 (11.8%)	3 (8.3%)	31 (86.1%)	2 (5.6%)
	Attending physician	126 (41.4%)	17 (13.5%)	100 (79.4%)	9 (7.1%)
	Associate chief physician	68 (22.4%)	11 (16.2%)	53 (77.9%)	4 (5.9%)
	Chief physician	74 (24.3%)	16 (21.6%)	52 (70.3%)	6 (8.1%)
		*X* ^2^	3.948	4.022	0.390
		*P*	0.267	0.259	0.942

### Physicians' evaluations of DPR

Physicians generally had a negative attitude toward DPR and perceived DPR as poor. A total of 84.5% (257/304) of physicians had a DDPRQ-8 score of 17 or higher, suggesting that they perceived DPR as poor or strained; this group included the 6.9% (21/304) of physicians with a DDPRQ-8 score of 33 or higher, suggesting that they perceived DPR as strained (Table 4).

**Table 4:** Physicians' evaluations of doctor–patient relationship.

**Perceived relationship**	**Number (*n*)**	**Percentage (%)**
Good	47	15.5
Poor	236	77.6
Strained	21	6.9

### Physicians' evaluations of DPR under demographic information

Physicians generally perceived DPR as poor under different demographic information, including age, gender, educational level and professional title (Table 2). There were no significant different of physicians' evaluations of good, poor or strained DPR, among different levels of age, gender, educational level and professional title, respectively (The values of χ*2* and *P* were shown in Table 3).

### Physicians' preferences for SDM and the possible reasons

A total of 53.3% (162/304) of physicians preferred SDM, while 21.1% (64/304) did not prefer SDM (Table 5).

**Table 5:** Physicians' preferences for shared decision-making.

**Level of propensity**		**Number (*n*)**	**Percentage (%)**
High	Considerably high	44	14.5
	Relatively high	118	38.8
Neutral	Neutral	78	25.7
Low	Relatively low	50	16.4
	Extremely low	14	4.6

An investigation of the possible reasons for physicians' preferences indicated that physicians preferred SDM mainly because of the influences of medical ethics and social desirability bias, with medical ethics accounting for 60.5% of the responses. The leading reasons for a neutral propensity were the same as above, with medical ethics accounting for 30.8% and social desirability bias accounting for 39.7%. The leading reasons for low propensity were poor DPR and low levels of medical knowledge among patients, with medical ethics accounting for 34.4% and social desirability bias accounting for 28.1% of the responses. The results of the chi-square test showed that the differences among physicians with different preferences for SDM were statistically significant (χ*2* = 124.445, *P* < 0.001) (Table 6).

**Table 6:** Reasons for different preferences for shared decision-making (SDM) [*n* (%)].

**Level of propensity**	**Reasons for preferences for SDM**					
	**Medical ethics**	**Social desirability bias**	**Doctor–patient relationship**	**Patients' willingness to participate in SDM**	**Patients' level of medical knowledge**	**Whether SDM is necessary for patient's conditions**
High	98 (60.5)	40 (24.7)	9 (5.6)^*^	6 (3.7)^*^	4 (2.5)^*^	5 (3.1)^*^
Neutral	24 (30.8)	31 (39.7)	5 (6.4)^*^	7 (9.0)^*^	5 (6.4)^*^	6 (7.7)^*^
Low	6 (9.4)^†^	5 (7.8)^†^	22 (34.4)	8 (12.5)^†^	18 (28.1)	5 (7.8)^†^

* P < 0.001 vs. medical ethics and social desirability bias;

† P < 0.001 vs. doctor–patient relationship and patients' level of medical knowledge.

### Impact of physicians' evaluations of DPR on their preferences for SDM

Spearman's rank correlation coefficient showed that physicians' evaluations of poor DPR and their preferences for SDM were independent of each other. There was no significant correlation between the two (*r* = −0.082, *P* = 0.155).

Fisher's exact test showed that similar proportions of physicians preferred SDM regardless of whether they perceived DPR as good, poor or strained; the respective proportions were 42.6, 56.4 and 42.9%. The preferences for SDM were not significantly different among physicians who perceived good, poor or strained DPR (*P* = 0.073) (Table 7).

**Table 7:** Preferences for shared decision-making (SDM) among physicians with different evaluations of doctor–patient relationship (DPR) [*n* (%)].

**Evaluation** **of DPR**	**Propensity toward SDM**			
	**High**	**Neutral**	**Low**	**Total**
Good	20 (42.6) ^*^	11 (23.4)	16 (34.0)	47 (100)
Poor	133 (56.4)^*^	58 (24.6)	45 (19.1)	236 (100)
Strained	9 (42.9) ^*^	9 (42.9)	3 (14.3)	21 (100)

* P = 0.073.

## Discussion

As a good clinical decision-making model, SDM has the following characteristics compared with the traditional informational mode and parental mode: physicians and patients both participate in decision-making, equally share information about disease diagnosis and treatment, establish consensus through continuous communication, and, on this basis, reach an agreement regarding the diagnosis and treatment plans (1, 30–33). SDM aims to reduce the differences and discrepancies between physicians and patients, create optimal treatment plans, reduce medical costs, conserve social resources, eliminate doubts, promote enthusiasm for compliance and self-management in patients, and facilitate mutual empathy (2, 4, 7, 34–43), which is of great significance and is the best way to build a medical service system in which physicians and patients act in accord (33, 44–46). Therefore, SDM should be the preferred decision-making model among physicians.

The frequency of medical disputes and violent acts against doctors in recent years deteriorated the DPR (25, 26), making doctors feel insecure and causing them to perceive poor DPR in their medical practice (47). The results of this study showed that physicians generally hold a negative attitude toward DPR, with up to 84.5% of physicians perceiving poor or strained DPR. As one of the main actors in DPR, physicians' evaluations of poor DPR will reduce their sense of professional identity and pride and will cause anxiety, depression, and other negative emotions (48); compromise their occupational satisfaction, sense of self-accomplishment, and subjective well-being; and reduce their investment in their daily work (49, 50). When patients feel that they are being neglected or treated indifferently, they inevitably become dissatisfied and resent their doctors, which exacerbates the already strained DPR and can even result in disputes, thus forming a vicious cycle (51–56). If the situation continues, it is bound to greatly frustrate the orientation of doctors and patients to each other and may affect patients' participation in SDM. To reduce medical risks, avoid medical disputes, and strengthen self-protection, doctors may engage in defensive medical behaviors (57–59), such as performing a wide range of laboratory tests, examinations and treatments; avoiding high-risk surgery and the admission of high-risk patients; providing unnecessary referral options and consultations to pass the buck; concealing the patient's true condition or exaggerating the conditions and the risk of treatment; and intentionally choosing conservative treatments for patients.

In this context, do physicians' evaluations of DPR affect their propensities for SDM in medical practice? A study of physicians' preferences and experiences in clinical decision-making showed that approximately 75% of physicians preferred SDM and that 87% believed that they applied preferred decision-making models (60). The results of this study also showed no overall correlation between physicians' evaluations of poor DPR and their preferences for SDM. Regardless of whether physicians perceived DPR as good or bad, their strong willingness to practice SDM was unaffected. This shows that physicians are fully aware of the importance of SDM; even if they were not optimistic about current DPR, most of them were willing to practice SDM. In the current situation of strained DPR, the strong propensity of physicians toward SDM strongly reflects their high awareness of medical ethics, strong sense of responsibilities and socially desirable behavior, and positive subjective intentions. This finding was validated by the analysis of the reasons why physicians prefer SDM. This result is in line with the principle of respect in medical ethics, which means that physicians and patients should respect each other sincerely in their communication and emphasizes that medical staff should respect patients and their families. That is to say, physicians are required to respect patients' personality rights and autonomy, so as to ensure patients' autonomy to make rational treatment decisions. The essence of independent choice is to respect and maintain the patients' independent rights. Respecting the principle is the necessary condition and reliable foundation to establish a harmonious DPR (61, 62). Poor DPR are the leading reason why physicians are unwilling to practice SDM; given the generally strong propensity toward SDM, poor DPR might affect the choice of only a small number of physicians.

Some scholars believe that the use of SDM can alleviate strained DPR (63–65). This study also found that among the 44 physicians with considerably high preferences for SDM, only 3 (6.8%) believed that DPR were strained, while among the 14 physicians who had extremely low preferences for SDM, 2 (14.3%) believed that DPR were strained, a proportion that was higher than that of the former group. Although a larger sample size is needed to verify this finding, the above data may suggest that SDM is conducive to building harmonious DPR. In future studies, we will expand the sample size, further investigate patients' evaluations of DPR, comprehensively evaluate the evaluations of DPR among physicians and patients and explore whether physicians' adoption of SDM will improve DPR.

Of course, it is worth mentioning that physicians' willingness to practice SDM is not the only factor in this decision-making model that is decisive in achieving desired results and reaching a consensus on diagnosis and treatment in clinical practice. Effective communication between physicians and patients is also a decisive factor, since patients are an important actor involved in SDM. Pollard et al. and Aoki et al. found that although physicians prefer SDM in clinical practice, physicians ultimately make decisions alone (34, 66). This finding indicates that in the clinical decision-making process, the decision-making model that physicians adopt is not always the one they claim to prefer. Although Chinese patients have a stronger intention to participate in decision-making, they are restricted by their educational levels, economic conditions, and medical knowledge (67). Moreover, patients' family members participate in decision-making regarding diagnosis and treatment more frequently than the patients do. Even though Chinese physicians prefer SDM, it is often difficult for them to engage in SDM in actual clinical practice due to the limited participation of patients.

The late start on protecting patients' right to informed consent and the slow promotion of the SDM concept in China is partially responsible for the current situation, while the lack of effective communication between physicians and patients is the leading factor. The modern medical model has changed from the traditional biomedical model to the biopsychosocial medical model. Patients' demands for medical healthcare are diverse, and their willingness to participate in treatment decisions has become increasingly strong. Physicians also perceive the strong willingness of patients and their families to participate in decision-making regarding diagnosis and treatment. Therefore, physicians are willing to practice SDM in their communications with patients. However, the high workload of physicians, insufficient time for communication, and the generally high expectations and requirements of diagnosis and treatment results among patients and their families impose enormous pressure on physicians. Therefore, physicians may fail to communicate with patients harmoniously and effectively in their attempts to practice SDM. Moreover, the rapid development and wide application of high-end medical technology in clinical practice enables physicians to obtain important objective information on patients' conditions using highly sensitive instruments and equipment. As a result, physicians may not be fully aware of patients' information needs. These problems are obstacles to SDM (68). Ineffective outcomes of SDM will result in information asymmetry between physicians and patients. Patients lack information on the efficacy, benefits, and risks of diseases and relevant treatment regimens, and their partial understanding results in unrealistic expectations regarding efficacy and prognosis. Physicians do not fully understand the values, preferences, and family conditions of patients. Under such situations, it is difficult for patients and physicians to cooperatively make the most suitable decisions for patients, which raises the potential risk of doctor–patient conflicts.

Therefore, although strained DPR generally did not affect physicians' preferences for SDM, it may reduce the willingness of some physicians to practice SDM and may affect the final outcomes of SDM. We will expand the sample size and conduct an in-depth investigation and analysis of this issue. Moreover, this study did not include the investigation of patients' evaluations of DPR. The unilateral physicians' evaluations could reflect the true psychological states of physicians DPR, but it might lead to less objective evaluation of DPR tension. We will investigate and compare the evaluations of physicians and patients of DPR in future research, so as to improve the real evaluation of DPR tension. In addition, quantitative assessment such as scores may be used to identify the possible reasons for physicians' evaluations of SDM instead of qualitative evaluation. We will quantify the evaluation method in future research.

## Conclusion

Most of physicians have poor evaluations of DPR, but it does not affect their choice tendency for SDM, which may be because of the influence of professional ethics and social desirability bias. Under the current environment of tense DPR, physicians have a strong willingness to make SDM to determine the treatment plan along with the participation of patients and their families.

## Data availability statement

The raw data supporting the conclusions of this article will be made available by the authors, without undue reservation.

## Ethics statement

Ethical review and approval was not required for the study on human participants in accordance with the local legislation and institutional requirements. The patients/participants provided their written informed consent to participate in this study.

## Author contributions

LL and Y-WC: conceptualization. Z-RC and Y-HL: data curation. LZ, M-YX, and Y-HL: formal analysis. LZ and Y-WC: investigation. LZ, Y-WC, M-YX, and HJ: project administration. LL: supervision and writing—review and editing. Y-WC: visualization. Z-RC and LZ: writing—original draft. All authors contributed to the article and approved the submitted version.

## Conflict of interest

Author Y-WC was employed by the company Genecast Biotechnology Co., Ltd. The remaining authors declare that the research was conducted in the absence of any commercial or financial relationships that could be construed as a potential conflict of interest.

## Publisher's note

All claims expressed in this article are solely those of the authors and do not necessarily represent those of their affiliated organizations, or those of the publisher, the editors and the reviewers. Any product that may be evaluated in this article, or claim that may be made by its manufacturer, is not guaranteed or endorsed by the publisher.
